# Fuzzy Logic-Based Geographic Routing Protocol for Dynamic Wireless Sensor Networks

**DOI:** 10.3390/s19010196

**Published:** 2019-01-07

**Authors:** Xing Hu, Linhua Ma, Yongqiang Ding, Jin Xu, Yan Li, Shiping Ma

**Affiliations:** 1 School of Aeronautics Engineering, Air Force Engineering University, Xi’an 710038, China; xuj504@163.com (J.X.); lywin2002@163.com (Y.L.); mashiping@126.com (S.M.); 2 Institute of Unmanned Systems Technology, Northwestern Polytechnical University, Xi’an 710072, China; Land_max@126.com; 3 Aviation Petty Officer School, Air Force Engineering University, Xinyang 464000, China; dingyongqiang654@163.com; 4 Xi’an Institute of Space Radio Technology, Xi’an 710000, China

**Keywords:** geographic routing protocol, fuzzy logic, selection criteria, parameters for assessment, routing overhead

## Abstract

The geographic routing protocol only requires the location information of local nodes for routing decisions, and is considered very efficient in multi-hop wireless sensor networks. However, in dynamic wireless sensor networks, it increases the routing overhead while obtaining the location information of destination nodes by using a location server algorithm. In addition, the routing void problem and location inaccuracy problem also occur in geographic routing. To solve these problems, a novel fuzzy logic-based geographic routing protocol (FLGR) is proposed. The selection criteria and parameters for the assessment of the next forwarding node are also proposed. In FLGR protocol, the next forward node can be selected based on the fuzzy location region of the destination node. Finally, the feasibility of the FLGR forwarding mode is verified and the performance of FLGR protocol is analyzed via simulation. Simulation results show that the proposed FLGR forwarding mode can effectively avoid the routing void problem. Compared with existing protocols, the FLGR protocol has lower routing overhead, and a higher packet delivery rate in a sparse network.

## 1. Introduction

As a novel information acquisition and processing mode, the wireless sensor network [1,2] collects information through a large number of sensor nodes distributed within a monitored area and then provides it to users after processing. Due to the limited communication distance between sensor nodes, information transmission needs to be completed by multi-hops. Therefore, a routing technology with low routing overhead and high reliability has become the key technology used for wireless sensor networks.

Geographic routing can establish routing by using only the location information of local nodes. Specifically, it can directionally forward information to a destination node without flooding the information in the entire network. Hence, geographic routing is considered very efficient in multi-hop wireless sensor networks.

For existing geographic routing protocol, it is generally assumed that nodes can obtain their own positions through location services [3] or a global position system (GPS) [4], and the location of a destination node can be acquired through a location service algorithm, such as the grid location service (GLS) [5].

However, with the application expansion of wireless sensor networks, the movement of sensor nodes will occur in some scenarios, such as aviation sensor networks. For dynamic wireless sensor networks, the existing routing protocols, based on location service algorithms, incur an additional routing overhead and storage overhead. In addition, the obtained node location is not accurate enough, resulting in routing failure. Consequently, the high routing overhead problem and inaccuracy location problem also occur in existing geographic routing protocols.

In addition, the routing void problem and network planarization problem [6] also occur for the main component of geographic routing (the greedy forwarding mode and perimeter forwarding mode [7]), resulting in a longer path with a higher overhead. 

This paper proposes a fuzzy logic-based [8,9] geographic routing protocol (FLGR) through researching the geographic routing protocol without the real-time location information of the destination node, and aims at solving the aforementioned problems. In particular, the main contributions of this paper are as follows:

- A novel data forward mode is proposed based on the fuzzy location region of the destination node.
- Aiming for fewer hops and a higher packet delivery rate, the selection criteria and parameters for assessment are also proposed.
- In addition, to avoid the routing void problem, the routing void avoidance scheme is proposed to reduce the rate of routing failure by adjusting the candidate node region (CNR) and discarding the void node.
- Based on the fuzzy logic system, subordinating degree functions, the fuzzy reasoning rule, and the defuzzification method are proposed.
- The feasibility of the FLGR forwarding mode is verified through MATLAB simulation. The performances between FLGR protocol and existing protocols are compared by simulation using the QualNet platform.

The rest of the paper is organized as follows: Section 2 presents previous works on this topic. Section 3 describes the details of the FLGR protocol. The feasibility verification of the FLGR forwarding mode and the performance comparison between FLGR protocol and others are presented in Section 4, followed by conclusions in Section 5.

## 2. Related Studies

In dynamic wireless sensor networks, the high routing overhead problem, routing void problem, and inaccuracy location problem occur frequently for geographic routing protocols. Hence, the data forwarding mode and location prediction algorithm of geographic routing have become research hotspots. Thus, in this paper, the fuzzy logic-based geographic routing protocol and location-fault-tolerant geographic routing scheme will be introduced.

A typical geographic routing protocol, greedy perimeter stateless routing (GPSR) is proposed in [7]. The greedy forwarding mode and peripheral forwarding mode are adopted in the GPSR protocol. In the greedy forwarding mode, the neighbor node nearest to the destination node is selected as the next forwarding node. However, the routing void problem occurs in a sparse network. The peripheral forwarding mode can solve this problem, based on planarization and right-hand rules.

Some improved data-forwarding strategies are also proposed. In Reference [10], a new GPSR protocol based on angle and distance (GPSR-AD) is proposed. In the GPSR-AD protocol, the distance and angle are considered in selecting the next forwarding node, which can effectively reduce the number of routing hops. Guan et al. put forward an improved greedy forwarding strategy based on fuzzy logic control [11]. The new scheme can actively choose the next forwarding node in order to reduce the end-to-end delay, stabilize the queue length, and improve transmitting efficiency. A congestion-aware forwarder selection method for greedy forwarding mode is proposed in Reference [12]. The energy consumption and end-to-end delay can be reduced by combining multiple performance metrics, including energy consumption, forwarding direction, and congestion level of a potential forwarder. To solve the routing failure, Yu and Ahn [13] proposed a geographic routing scheme that can provide reliable and efficient routes by computing the range of azimuth angles that it can cover.

There are many existing studies that explore location prediction-based and location-fault-tolerant geographic routing protocol [14,15,16,17]. Based on the autoregressive integrated moving average (ARIMA) prediction model [18], the optimal path for data transmission can be selected to reduce the routing reconstruction overhead [14,15]. In Reference [16], Wang proposes an efficient geographic routing scheme based on location prediction and energy saving (LPESGR). Based on the prediction distance of signal transmission, the routing void problem can be solved by a new power real-time adjustment scheme. The LPESGR protocol can effectively reduce network energy consumption and improve the success rate of data transmission. To resolve the location inaccuracies problem, Lin et al. proposed a novel location-fault-tolerant geographic routing (LGR) [17] scheme based on the combination of position-based clustering and geographic routing technologies.

Although studies on data forwarding strategies and the node location prediction algorithm can reduce the number of hops and the forwarding delay to some extent, existing strategies tend to increase the extra routing overhead to obtain the location information of destination nodes through the location service algorithm. Especially in dynamic wireless sensor networks, the location information of destination nodes should be acquired in a timely and accurate manner, which would inevitably increase the routing overhead.

## 3. FLGR Protocol

The traditional data forwarding mode is not suitable in the situation without the accuracy location information of the destination node. In FLGR protocol, the nodes to be selected can first be confirmed, based on the fuzzy location region of the destination node. Then, we can select the better forwarding node based on the fuzzification, fuzzy reasoning, and defuzzification of node parameters. In addition, the scheme to avoid the routing void problem is also proposed. In this way, the FLGR protocol can effectively reduce routing overhead and improve the success rate of data transmission.

### 3.1. Network Model and Assumptions

Consider a wireless sensor network with *N* nodes, which move randomly in the region with an area of *S*. In this paper, $v_{\max}$ denotes the maximum speed of a node, and R denotes the transmission radius of a node. Each node is equipped with a GPS receiver, which can obtain its location information. Each node periodically broadcasts a “Hello” message, which contains the location information of themselves and their neighbor nodes. Each node knows its own neighbor nodes through an exchanging beacon.

In this study, the following assumptions are made for the network model: (1) A two-dimensional space is considered, and $l_{i}$ denotes the location information of node $n_{i}$, while the coordinates of node i can be denoted by $\left(x_{i},y_{i}\right)$. (2) We assume that the geographical locations of any two nodes do not overlap, that is, if ∃i≠j, then $l_{i}\neq l_{j}$. (3) When a network is established, each node stores the initial location information of all the network nodes locally. (4) There is no error in data transmission within the range of transmission. A single data packet can be transmitted completely, without considering the transmission link interruption. (5) Each node in the network maintains time synchronization.

For ease of description, a detailed list of acronyms and notations adopted in this section is presented in Table 1.

### 3.2. Message Format and Local Storage Structure

The Hello message is the basis of the FLGR protocol. Based on Hello messages, the neighbor nodes can be found, and their location information can be obtained. The format of the Hello message is presented in Table 2.

As given in Table 2, each Hello message has a unique sequence number, Num_seq, which can be used to indicate the freshness of the information carried by the Hello message. The ID of the Hello message denotes the unique identity label of the sending node. The neighbor node IDs and locations are also included in the Hello message, which can be used to obtain the parameter of CN. The node location can be represented by 16 Bytes. The x and y axis can be represented by 64 bits, respectively, with 1 bit to the sign of the number, 16 bits to the exponent, and 47 bits to the fractional part. Through the periodic broadcasting of the Hello message, the information table of neighbor nodes can be established.

To update the neighbor node table in a timely manner, the periodicity of the Hello message changes according to the node’s maximum speed. In Section 4.2, the periodicity of the Hello message is given in different scenarios.

When the Hello message reaches a node, the neighbor node table will be updated. The neighbor node will be deleted in neighbor node table when its information is not included during two Hello message intervals. Once the information of a new node occurs in the Hello message, it will be added to the neighbor node table.

In addition, other than the transmission data, the data message also contains information on the destination node and all previous forwarding nodes. The format of the data message is given as follows:

As given in Table 3, Num_seq indicates the freshness of the information carried by the data message. Node IDs_pre denotes the IDs of all previous forwarding nodes. Nodes locations_pre denotes the locations of all previous forwarding nodes. Num_pre denotes the number of all previous forwarding nodes. In practical application, the maximum value of Num_pre should be set. This changes according to different scenarios. If the reserve space is full, the latter forwarding node information is discarded.

With the transmission of data messages in the network, each node can update its local network location table frequently. The local network location table is presented as follows:

As shown in Table 4, each node stores the local network location table, including node ID, node location, and update time. The update time indicates when the last update of the node location occurs. If a node has a message to be sent, the fuzzy region of the destination node can be obtained by the interval between the update time of the destination node in the local network location table and the current moment (as shown in Formula (1)).

### 3.3. FLGR Forwarding Mode

Owing to the inaccurate location of the destination node, the greedy forwarding mode does not fit into the FLGR protocol. The next forwarding node can be selected by the node parameters of CN. Taking an example in which EN $n_{1}$ sent a data message to the destination node $n_{8}$, the FLGR forwarding mode is shown as follows.

**Figure 1:**
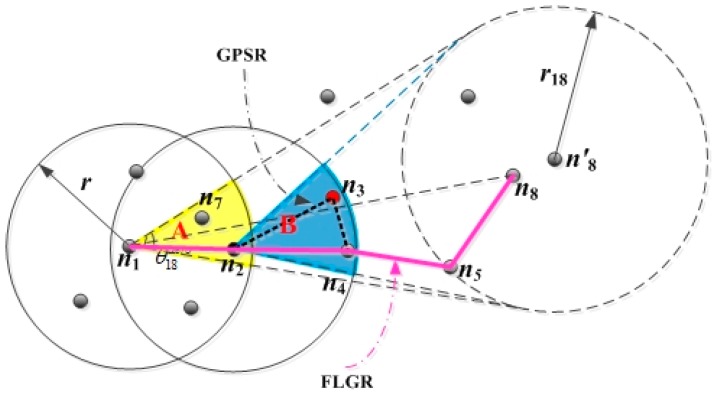
Fuzzy logic-based geographic routing protocol (FLGR) forwarding mode.

As shown in Figure 1, the location of node $n_{8}$ is the current accuracy location, and the location of node $n_{8}^{\prime }$ is the fuzzy location by looking up the local network location table of EN $n_{1}$. When EN $n_{1}$ has a data message to be sent to the destination node $n_{8}$, the previous location and update time of node $n_{8}$ can be obtained through the local network location table of EN $n_{1}$. Next, the fuzzy location region of node $n_{8}$ can be obtained, according to the time interval $t_{18}$ from the previous update time to the current moment. The fuzzy location region can be denoted by radius $r_{18}$, which can be calculated as follows:

(1) $$r_{18}=t_{18}*v_{\max}$$

According to the fuzzy location region of destination node $n_{8}$, we can get the CNR A of the EN $n_{1}$. The CNR A is denoted by angle $\theta_{18}^{aero}\in (0,\pi )$, which can be calculated as follows:

(2) $$\theta_{18}^{aero}=2\times \arccos\frac{r_{18}}{{\left\|l_{1}-l_{8}\right\|}_{2}}$$

To select the better forwarding node in the CNR of EN $n_{1}$, a comprehensive assessment of node $n_{2}$ and $n_{7}$ should first be obtained.

### 3.4. Selection Criteria and Parameters for Assessment

To obtain a comprehensive assessment of the nodes in CNR, the selection criteria and parameters for assessment should be confirmed first.

Fewer hops and a higher packet delivery rate are the goals of the FLGR forwarding mode. In addition, the scheme used to avoid the routing void problem should also be considered. Based on this, this paper gives the selection criteria of the next forwarding node:

- The CN has a larger probability of being selected if it is farther away from EN. That is, a larger distance between the EN and CN indicates that the probability that the CN is close to the destination node is larger.
- The CN that has more nodes in its CNR has a larger probability of being selected to be the next forwarding node. If there are more nodes to be selected in the CNR, the probability of selecting the optimal forwarding node is larger in the next data forwarding node. In this way, the node that has no CN in its CNR will be discarded, and thus, the routing void problem can be solved.
- The CN has an advantage in being selected to be the next forwarding node if its CNs distribute more evenly along the communication boundary.

According to the above selection criteria, the void node can be avoided effectively. As shown in Figure 1, in the GPSR protocol, according to the greedy forwarding mode and peripheral forwarding mode, the nodes $n_{2},n_{3},n_{4},n_{5}$ are selected as forwarding nodes. The nodes $n_{2},n_{4},n_{5}$ are selected as forwarding nodes according to the selection criteria of the FLGR protocol. In this way, the void node $n_{3}$ can be discarded, and the number of hops reduces. Hence, the routing void problem can be effectively avoided by using the selection criteria above.

According to the selection criteria of the FLGR protocol, the corresponding parameters of CN can be obtained: the distance to EN, relative density of the nodes in CNR, and distribution degree of the nodes in CNR.

Distance to EN is the distance between CN and EN, which is denoted by D in this paper. The value range of D is (0,r]. Taking no account of link interruption, the larger D is, the more likely CN is to approach the destination node. In this paper, the set $C_{i}$ denotes the set of nodes in CNR of EN. For node $n_{4}\in C_{i}$, the distance between node $n_{k}$ and EN $n_{i}$ can be calculated as follows:

(3) $$D_{ik}=\{\begin{array}{ll} {\left\|l_{k}-l_{i}\right\|}_{2} & ,C_{i}\neq \emptyset \\ 0 & ,C_{i}=\emptyset \end{array}$$

Relative density of the nodes in CNR is the ratio of the density of nodes in CNR of CN to the density of the entire network, which is denoted by η. In this paper, the set $Z_{k}$ denotes the set of nodes in the CNR of CN. If $\exists n_{v}\in C_{i}$, then $n_{v}\notin Z_{k}$. In this way, invalid routing can be effectively avoided.

In FLGR protocol, a larger value of η indicates that there are more nodes in CNR. The probability of selecting the optimal forwarding node is larger with more nodes to be selected. Taking the example of CN $n_{k}$, when EN $n_{i}$ has a data message to be sent to the destination node $n_{j}$, there are m neighbor nodes in the CNR of CN $n_{k}$. Then, $\eta_{kj}$ can be expressed as follows:

(4) $$\eta_{kj}=\frac{\rho_{kj}}{\rho_{global}}=\frac{2m*S}{n*r^{2}*\theta_{ij}^{aero}}$$

In Equation (4), $\rho_{kj}$ denotes the density of nodes in the CNR of CN $n_{k}$. $\rho_{global}$ denotes the density of the entire network. The value range of η is [0,+∞). An increase in the value of η, indicates that the density of the nodes in CNR increases, and the number of nodes is relatively bigger. However, the case of η>9 is a small probability event according to statistics in the simulation in Section 4. Therefore, the situation η∈[0,9] is considered in this paper.

The distribution degree of the nodes in CNR represents the distribution of nodes in the CNR of CN, and it is denoted by Ω in this paper. In the FLGR protocol, if the nodes in CNR are evenly distributed along the communication boundary, the distribution of the nodes in CNR is optimal. The probability that the CN is selected as the next forwarding node is the highest. However, if the nodes in CNR are centrally distributed near the CN, the distribution of the nodes in CNR is the worst. Taking the CN $n_{2}$ of EN $n_{1}$, for example, node $n_{4}$ and $n_{5}$ are in the CNR of CN $n_{2}$, and the optimal distribution and worst distribution are shown as follows.

**Figure 2:**
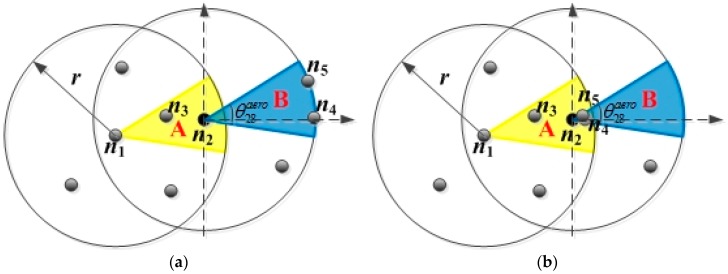
Distribution of the nodes in the CNR of node $n_{2}$: (**a**) Optimal distribution; (**b**) worst distribution.

As shown in Figure 2a, there are two nodes ($n_{4}$ and $n_{5}$) in the CNR B of CN $n_{2}$, and they are uniformly distributed along the communication boundary. In this situation, the parameter Ω of CN $n_{2}$ is the largest. By contrast, the distribution of nodes $n_{4}$ and $n_{5}$ in Figure 2b is the worst, and the parameter Ω of CN $n_{2}$ is smallest.

Thus, taking the CN $n_{k}$ for example, if $Z_{k}\neq \emptyset$, and there are m nodes in its CNR, the optimal locations of the m nodes, denoted by $L^{opti\_kj}$, can be obtained as follows:

(5) $$l_{u}^{opti\_kj}=\left(x_{k}+\cos(\frac{\theta_{kj}^{aero}*u}{m+1}+(\theta_{kj}-\frac{\theta_{kj}^{aero}}{2})),y_{k}+\sin(\frac{\theta_{kj}^{aero}*u}{m+1}+(\theta_{kj}-\frac{\theta_{kj}^{aero}}{2}))\right),m\geq 1$$

As shown in Equation (5), taking the node $n_{k}$ as the origin of coordinates, the angle $\left(\frac{\theta_{kj}^{aero}*u}{m+1}+(\theta_{kj}-\frac{\theta_{kj}^{aero}}{2})\right)$ represents the angle of the uth optimal distribution node relative to the CN $n_{k}$.

In this paper, the distribution function is defined as $f(L,L^{opti},m)$, and the parameter $\Omega_{kj}$ of CN $n_{k}$ can be obtained as follows:

(6) $$\Omega_{kj}=f(L,L^{opti},m)=\frac{\sum_{i=1}^{m}\overset{m}{\underset{j=1}{\min}}{\left({\left\|l_{i}-l_{j}^{opti}\right\|}_{2}\right)}^{2}}{m*R^{2}},m=1,2,\ldots$$

In Equation (7), $L=(l_{1},\ldots ,l_{m})$ are the positions of m modes in CNR. According to the distribution function, the value range of parameter Ω is (0,1].

### 3.5. Selection Process Based on Fuzzy Logic

Owing to the uncertainty and no boundary of node parameters, it is very difficult to find the optimal weight coefficient for the decision algorithm based on utility function [19] and multiple-attribute decision-making [20]. The decision algorithm based on machine learning and game theory [21] has a higher complexity and longer time delay. Therefore, the decision algorithm based on fuzzy logic is chosen in this paper, owing to its higher accuracy and lower complexity. In addition, the decision algorithm based on fuzzy logic can express the expression of the human beings accurately. The decision algorithm based on fuzzy logic involves fuzzification, fuzzy reasoning and defuzzification.

#### 3.5.1. Fuzzification of Node Parameters

Fuzzification mainly refers to the fuzzification of attribute parameters through the subordinating degree function, to obtain the fuzzy set corresponding to the attribute. In general, the commonly used subordinating degree functions include the triangular subordinating degree function, trapezoidal subordinating degree function, sigmoid subordinating degree function and Gaussian subordinating degree function. As Gaussian subordinating degree function can satisfactorily show the gradual change characteristics of intermediate transitional nature and meet the human thinking mode well, it is selected in the fuzzification stage. The Gaussian subordinating degree function is denoted by μ(x) in this paper.

In the FLGR protocol, parameter D represents the distance between the CN and the EN, which can be divided into three fuzzy sets, namely, “near, middle and far”. In this paper, the subordinating degree function of parameter D is as follows:

(7) $$\mu_{D\_near}(x)=\{\begin{array}{ll} 0,\;x\leq 0 & or\;x>r\\ e^{\frac{x^{2}}{2500}}, & 0<x\leq r \end{array}$$

(8) $$\mu_{D\_middle}(x)=\{\begin{array}{ll} 0,\;x\leq 0 & or\;x>r\\ e^{\frac{{\left(x-r/2\right)}^{2}}{2500}}, & 0<x\leq r \end{array}$$

(9) $$\mu_{D\_far}(x)=\{\begin{array}{ll} 0,\;x\leq 0 & or\;x>r\\ e^{\frac{{\left(x-r\right)}^{2}}{2500}}, & 0<x\leq r \end{array}$$

**Figure 3:**
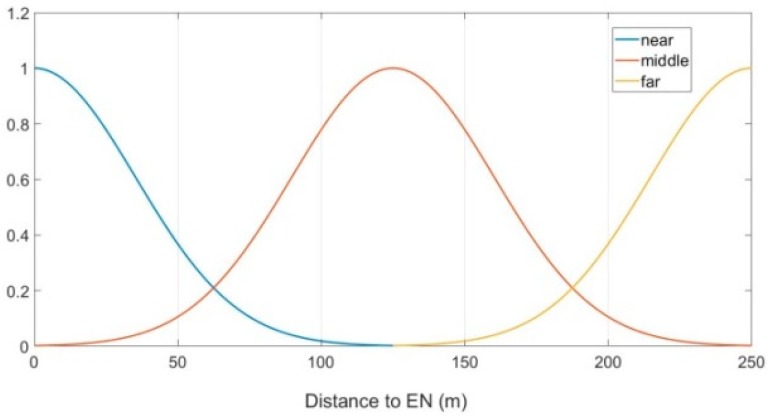
Subordinating degree function of parameter D with r=250.

Taking a communication radius of 250 m for example, Figure 3 shows the subordinating degree function of parameter D.

In the FLGR protocol, parameter η represents the intensive degree of the nodes in CNR, which can be divided into three fuzzy sets, namely, “low, middle and high”. In this paper, the subordinating degree function of parameter η is as follows:

(10) $$\mu_{\eta \_low}(x)=\{\begin{array}{ll} 0,\;x<0 & or\;x>9\\ e^{\frac{x^{2}}{4}}, & 0\leq x\leq 9 \end{array}$$

(11) $$\mu_{\eta \_middle}(x)=\{\begin{array}{ll} 0,\;x<0 & or\;x>9\\ e^{\frac{{\left(x-4.5\right)}^{2}}{4}}, & 0\leq x\leq 9 \end{array}$$

(12) $$\mu_{\eta \_high}(x)=\{\begin{array}{ll} 0,\;x<0 & or\;x>9\\ e^{\frac{{\left(x-9\right)}^{2}}{4}}, & 0\leq x\leq 9 \end{array}$$

**Figure 4:**
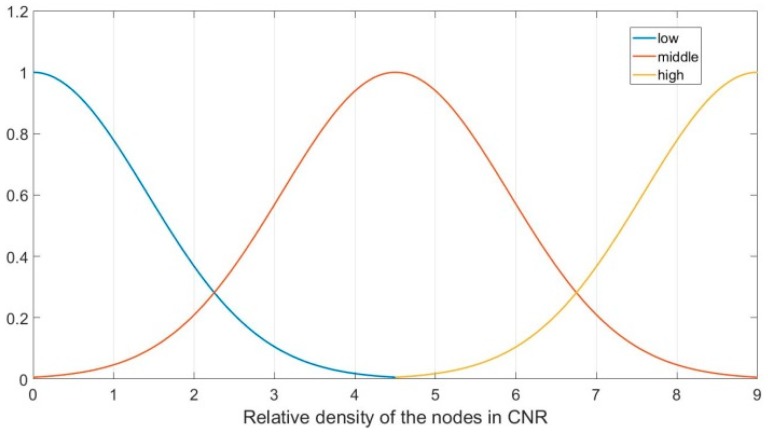
Subordinating degree function of parameter η.

Figure 4 shows the subordinating degree function of parameter η.

The parameter Ω represents the distribution situation of the nodes in the CNR of CN, which can be divided into three fuzzy sets, namely, “bad, middle, and excellent”. In this paper, the subordinating degree function of parameter Ω is as follows:

(13) $$\mu_{\Omega \_excellent}(x)=\{\begin{array}{ll} 0,\;x\leq 0 & or\;x>1\\ e^{\frac{x^{2}}{0.04}}, & 0<x\leq 1 \end{array}$$

(14) $$\mu_{\Omega \_middle}(x)=\{\begin{array}{ll} 0,\;x\leq 0 & or\;x>1\\ e^{\frac{{\left(x-0.5\right)}^{2}}{0.04}}, & 0<x\leq 1 \end{array}$$

(15) $$\mu_{\Omega \_bad}(x)=\{\begin{array}{ll} 0,\;x\leq 0 & or\;x>1\\ e^{\frac{{\left(x-1\right)}^{2}}{0.04}}, & 0<x\leq 1 \end{array}$$

**Figure 5:**
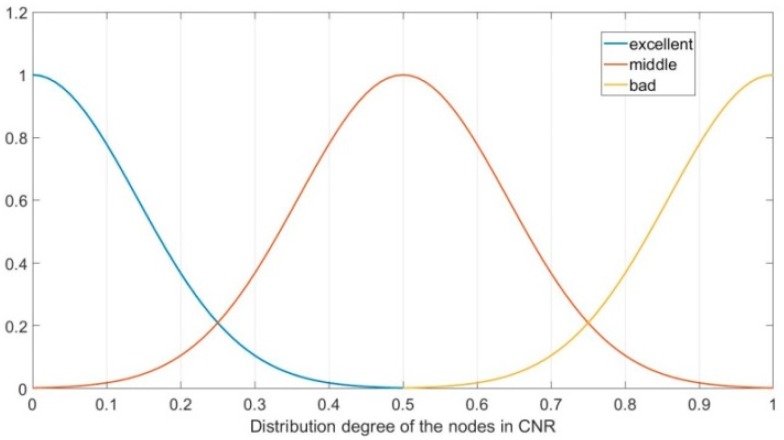
Subordinating degree function of parameter Ω.

Figure 5 shows the subordinating degree function of parameter Ω.

#### 3.5.2. Fuzzy Reasoning Rules and Fuzzy Reasoning Rule Base

In the fuzzy logic system, node parameter D, η and Ω are the input variables, and we can get the comprehensive assessment of CN as the output variable, which can be denoted by E. In this paper, five fuzzy sets (very low, low, middle, high, and very high) represent that the comprehensive assessment is very low, low, middle, high, very high, respectively. Therefore, there are a total of 3×3×3=27 fuzzy reasoning rules in the fuzzy reasoning rule base. A part of fuzzy reasoning rule base is presented as follows (The whole table is presented in Appendix A):

As given in Table 5, taking the node with the ID of 2 for example, its parameter D belongs to the middle fuzzy set, parameter η belongs to the middle fuzzy set, and parameter Ω belongs to the middle fuzzy set, as a result, the output parameter E belongs to the middle fuzzy set through the fuzzy logic system.

#### 3.5.3. Defuzzification

Through the two stages in fuzzy logic system above, the comprehensive assessments of all CNs can be obtained. However, when selecting the next forwarding node from the CNs, it is generally necessary to sort and compare the comprehensive assessments of all CNs. Hence, the comprehensive assessment of CN should be expressed by numerical values. Therefore, the defuzzification process should be conducted next.

Defuzzification transforms the fuzzy set into certain determined values, to conduct the subsequent mathematical calculation. The subordinating degree function of parameter E is as follows:

(16) $$\mu_{E\_very\;low}(x)=\{\begin{array}{ll} 0,\;x<0 & or\;x>1\\ e^{\frac{x^{2}}{0.01}}, & 0\leq x\leq 1 \end{array}$$

(17) $$\mu_{E\_low}(x)=\{\begin{array}{ll} 0,\;x<0 & or\;x>1\\ e^{\frac{{\left(x-0.25\right)}^{2}}{0.01}}, & 0\leq x\leq 1 \end{array}$$

(18) $$\mu_{E\_middle}(x)=\{\begin{array}{ll} 0,\;x<0 & or\;x>1\\ e^{\frac{{\left(x-0.5\right)}^{2}}{0.01}}, & 0\leq x\leq 1 \end{array}$$

(19) $$\mu_{E\_high}(x)=\{\begin{array}{ll} 0,\;x<0 & or\;x>1\\ e^{\frac{{\left(x-0.75\right)}^{2}}{0.01}}, & 0\leq x\leq 1 \end{array}$$

(20) $$\mu_{E\_veryhigh}(x)=\{\begin{array}{ll} 0,\;x<0 & or\;x>1\\ e^{\frac{{\left(x-1\right)}^{2}}{0.01}}, & 0\leq x\leq 1 \end{array}$$

**Figure 6:**
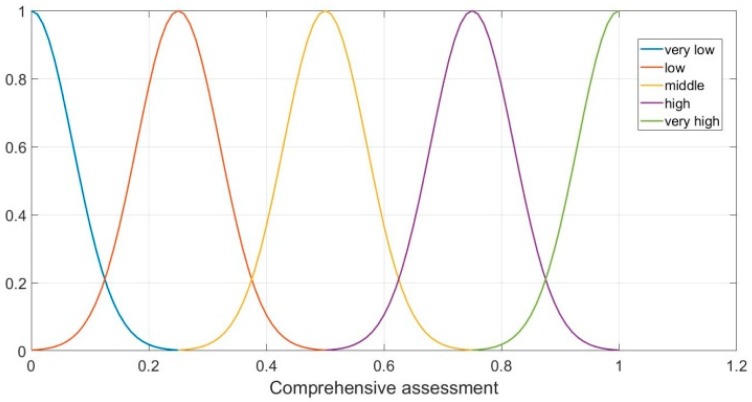
Subordinating degree function of parameter E.

Figure 6 shows the subordinating degree function of parameter E:

In the defuzzification stage, the graded mean integration representation method (GMIRM) [22] is adopted. The graded mean integration evaluation P(x) of fuzzy number x is as follows:

(21) $$P(x)=\frac{\int_{0}^{1}\left(\frac{h\left(2\delta -\sqrt{-\sigma_{L}{}^{2}\times \ln h}+\sqrt{-\sigma_{R}{}^{2}\times \ln h}\right)}{2}\right)dh}{\int_{0}^{1}hdh}$$

In Equation (21), $\sigma_{L}$ denotes the standard deviation of the left half of normal function, $\sigma_{R}$ denotes the standard deviation of the right half of normal function, and δ is the mean value of normal function.

In FLGR, when the fuzzy number x belongs to the very low set, the graded mean integration evaluation P(x) can be further simplified as follows:

(22) $$P(x)=\frac{\int_{0}^{1}\left(\frac{h\left(\delta +\sqrt{-\sigma_{R}{}^{2}\times \ln h}\right)}{2}\right)dh}{\int_{0}^{1}hdh}$$

When the fuzzy number x belongs to the very high set, the graded mean integration evaluation P(x) can be further simplified as follows:

(23) $$P(x)=\frac{\int_{0}^{1}\left(\frac{h\left(\delta -\sqrt{-\sigma_{L}{}^{2}\times \ln h}\right)}{2}\right)dh}{\int_{0}^{1}hdh}$$

When the fuzzy number x belonged to the low, medium and high set, $\sigma_{L}=\sigma_{R}$, and the graded mean integration evaluation P(x) can be further simplified as follows:

(24) P(x)=δ

Through the defuzzification by GMIRM, the comparison table between the fuzzy sets and their corresponding values can be obtained as presented in Table 6:

### 3.6. Routing Void Avoidance Scheme and Routing Process

Based on the fuzzy logic system, the next forwarding node can be selected and the route to the destination node can be established.

#### 3.6.1. Routing Void Avoidance Scheme

In the FLGR protocol, the routing void avoidance scheme is also adopted to solve the routing void problem. The routing void problem occurs when there is no CN (C=∅) in the CNR of EN, or there is no node (Z=∅) in the CNR of all CNs. In these situations, the next forwarding node can not be selected, so routing failure occurs.

To solve the routing void problem, the angle $\theta_{}^{aero}$ of CNR should be adjusted, which is obtained by the fuzzy location region of destination node. If there is no CN in the range of angle $\theta_{}^{aero}$, the angle will increase linearly and the CNR is expanded gradually until nodes occur in CNR. The data message will be discarded, if there is still no node in CNR until the angle $\theta_{}^{aero}$ reaches π. In this paper, ${\hat{\theta }}_{}^{aero}$ denotes the angle growth base, and it can be obtained as follows:

(25) $${\hat{\theta }}_{}^{aero}=k\times \frac{\theta^{aero}}{\rho_{global}}$$

**Figure 7:**
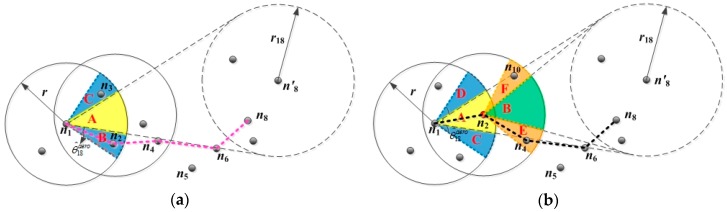
Routing void avoidance scheme: (**a**) In the situation of $C_{1}=\emptyset$; (**b**) in the situation of $C_{1}\neq \emptyset \cap Z_{2}=\emptyset$.

As shown in Equation (25), ${\hat{\theta }}_{}^{aero}$ is directly proportional to angle $\theta_{}^{aero}$, and it is inversely proportional to the entire network density $\rho_{global}$. k denotes the regulatory factor. Taking node $n_{1}$ as an example, the routing void avoidance scheme is shown in Figure 7:

As shown in Figure 7a, the routing void avoidance scheme operates when there is no node in the CNR A of EN $n_{1}$. The CNR A expands with the angle $\theta_{18}^{aero}$, increasing linearly by ${\hat{\theta }}_{18}^{aero}$ until nodes $n_{2}$ and $n_{3}$ occur. Then, according to the selecting process of the FLGR protocol, nodes $n_{2}$, $n_{4}$, and $n_{6}$ are selected as the next forwarding nodes.

Similarly, as shown in Figure 7b, only one $n_{2}$ exits in the CNR of EN $n_{1}$, and there is no node in the CNR of CN $n_{2}$. According to the routing void avoidance scheme, the regions A and B expand with the angle $\theta_{18}^{aero}$, increasing linearly by ${\hat{\theta }}_{18}^{aero}$, until the nodes $n_{4}$ and $n_{10}$ occur. Then, according to the selection process of the FLGR protocol, nodes $n_{2}$, $n_{4}$, and $n_{6}$ are selected as the next forwarding nodes.

#### 3.6.2. Routing Process

In the FLGR protocol, neighbor information can be maintained by periodically sending Hello messages. Taking the node $n_{x}$ for example, the key processing steps are as follows:

Step 1: Once node $n_{x}$ receives a data message, it updates its local network node location table according to the location information of the destination node and all previous forwarding nodes in the data message. If node $n_{x}$ is the destination node, the data message will be processed in the application layer, and the routing ends. Otherwise, go to step 2.
Step 2: Looking up the neighbor information of node $n_{x}$, if the destination node is its two-hop neighbor node, the next forwarding node can be selected according to the greedy forwarding mode. Otherwise, the updated location information of the destination node and node $n_{x}$ will be added to the data message. The CNR and CNs can be determined by the updated location information of the destination node. If the CNR angle of node $n_{x}$ is more than π ($\theta^{\mathrm{aero}}\geq \pi$), then go to step 7. Otherwise, go to step 3.
Step 3: If the CN set of node $n_{x}$ is empty ($C_{x}=\emptyset$), or $C_{x}\neq \emptyset$, but there is no node in the CNR of all CNs of node $n_{x}$ (Z=∅), go to step 6. Otherwise, go to step 4.
Step 4: For CNs with Z≠∅, the comprehensive evaluation values can be obtained through the fuzzification, fuzzy reasoning, and defuzzification of parameters (D, η, Ω). In addition, the CNs with Z=∅ are discarded. Then, go to step 5.
Step 5: The next forwarding node can be selected by ranking the comprehensive evaluation values of CNs. If multiple nodes have the best comprehensive evaluation value, the next forwarding node can be selected from them randomly.
Step 6: Operate the routing void avoidance scheme, and angle $\theta^{\mathrm{aero}}$ will increase linearly by ${\hat{\theta }}^{\mathrm{aero}}$ until nodes occur in the CNR. If the adjusted angle is more than π, go to step 7; otherwise, go to step 3.
Step 7: Discard the data message.

## 4. Simulation and Complexity Analysis

### 4.1. Feasibility Simulation of FLGR Forwarding Mode

To verify the feasibility of the FLGR forwarding mode, the performance of the FLGR forwarding mode and GPSR forwarding mode were compared using MATLAB. During simulation, the Random Way Point model was adopted, and 20, 60, 100, and 140 nodes were placed randomly in the two-dimensional plane of 1000 m×1000 m. The maximum speed of the node was set as 10 m/s, 20 m/s, 30 m/s, and 40 m/s, respectively, and the communication radius was set as 250 m.

Because the packet transmission process cannot be simulated in the MATLAB simulator, the time interval (100 ms) was adopted to represent the packet transmission process. For simplicity, the simulation starting time, randomly selected in [0, 3 s], is used to represent the time interval in the FLGR forwarding mode. $h_{F}$ and $h_{G}$ denote the numbers of routing hops in the FLGR and GPSR forwarding modes, respectively. $flag_{F}$ and $flag_{G}$ denotes the flags of the FLGR and GPSR forwarding modes, respectively. Taking the example of $n=60,v_{\max}=20\;m/s$, details of the simulation process are shown as follows:

Step 1: 60 nodes are placed randomly in the two-dimensional plane of 1000 m×1000 m. The location information is stored as an initial local network location table. Two nodes are randomly selected as a pair of transceivers. $h_{F}$, $h_{G}$, $flag_{F}$, and $flag_{G}$ are initialized to 0. Next, you move on to step 2.
Step 2: The simulation starting time is randomly selected in [0, 3 s]. It represents the time interval in formula 1. For the FLGR forwarding mode, according to the random time interval and initial local network location table, the fuzzy region of the destination node can be obtained using formula 1 and formula 2. Next, you move on to step 3 and step 4.
Step 3: If $flag_{F}=0$, according to the node selection algorithm of the FLGR forwarding mode, the next forwarding node can be selected, then $h_{F}$ adds 1, and go to step 4. Otherwise, go to step 5. If $flag_{G}=0$, according to the node selection algorithm of the GPSR forwarding mode, the next forwarding node can be selected, then $h_{G}$ adds 1, and go to step 4. Otherwise, go to step 5.
Step 4: If the packet reaches the destination node in the FLGR forwarding mode, $flag_{F}=1$; otherwise, all nodes move randomly in 100 ms with a speed of [0, 20 m/s]. Similarly, if the packet reaches the destination node in the GPSR forwarding mode, $flag_{G}=1$; otherwise, all nodes move randomly in 100 ms with a speed of [0, 20 m/s]. Then go to step 3.
Step 5: Routing process finishes.

**Figure 8:**
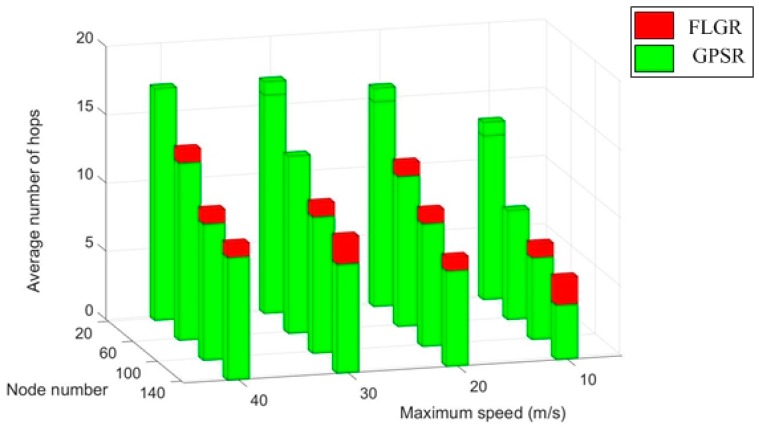
Comparison of average number of hops between FLGR and greedy perimeter stateless routing (GPSR).

To ensure the accuracy of the results, the average value of 1000 trials is rounded as the final result. The seed changes in every trial. In this simulation, the location information of the destination node is assumed to be known. The routing hops for obtaining the location information of the destination node are not included for the GPSR forwarding mode. The simulation results are shown in Figure 8.

As shown in Figure 8, with a certain node number, the average number of hops increases with an increase in maximum speed for the GPSR forwarding mode, as well as the FLGR forwarding mode. This phenomenon occurs because the network topology changes faster with the increase of node movement speed. For the GPSR forwarding mode, the optimal forwarding node is difficult to find, leading to more hops. Additionally, because the fuzzy location region of the destination node becomes larger, the average number of hops increases under the FLGR mode.

Under a certain maximum node speed, the average number of hops decreases with a increase in node number for both the forwarding modes. This phenomenon occurs because there are fewer and fewer void nodes with an increase in node number. For the GPSR forwarding mode, the probability of turning to the peripheral forwarding mode is small; thus, the number of hops decreases. For the FLGR forwarding mode, the probability of selecting the optimal forwarding node increases, because nodes in CNR increases with the increase in node number.

In addition, when the network is sparse (n=20), the average number of hops under the FLGR forwarding mode is less than that under the GPSR mode. This phenomenon occurs because there are more void nodes in the sparse network. For the GPSR forwarding mode, the probability of turning to the peripheral forwarding mode becomes large, resulting in more hops. For the FLGR forwarding mode, the void nodes can be discarded through the routing void avoidance scheme.

### 4.2. Comparison of Network Performance

To compare the network performances between the FLGR protocol and existing protocols, the QualNet network simulation platform was adopted to construct a simulation environment. The QualNet simulator is the only parallel and message-level network simulation tool, and was developed by Scalable Networks Technologies. It has a faster running speed, higher accuracy, and better extensibility, and is suitable for the development and simulation of wireless sensor networks.

During simulation, network nodes are randomly distributed in a two-dimensional plane of 1000 m×1000 m, where the random waypoint (RWP) movement model is adopted. Every node starts at an initial location and moves toward a randomly chosen destination location with a constant speed, which is randomly chosen in $\left[0,v_{\max}\right]$. This process repeats throughout the simulation, and we assumed that the pausing time was equal to zero in Section 4.2. Each node is equipped with a semi-duplex transceiver. The IEEE 802.11 protocol was selected for the physical layer as well as the MAC layer, where the transmission radius of the node was 250 m, and packets arrived to the node according to a Poisson process with 2packets/s; that is, an average of two packets arrived to each node per second. The GPSR, GPSR-AD, LPESGR, and FLGR protocols were adopted for the routing layer. For the GPSR, GPSR-AD, and LPESGR protocols, the GLS algorithm was adopted to obtain the location information of the destination node. According to different maximum speeds of the nodes (10, 15, 20, 25, 30, 35, 40 m/s), the frequencies of the Hello message were set to 1 Hz, 1.5 Hz, 2 Hz, 2.5 Hz, 3 Hz, 3.5 Hz, and 4 Hz respectively. The final data was obtained through the average of 8 trails, and the simulation time of each trail was 10 min. The detailed network parameters are presented in Table 7.

#### 4.2.1. Packet Delivery Ratio

The packet delivery ratio of the network refers to the ratio of the number of data packets received by the destination node to that sent by the source node, which reflects the reliability of the routing protocol.

**Figure 9:**
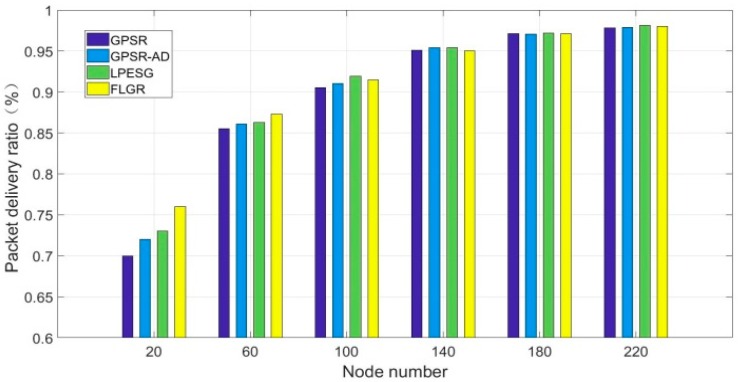
Impact of node number on packet delivery ratio with $v_{\max}=40$.

Figure 9 shows the impact of node number on the packet delivery ratio in a quickly changing network ($v_{\max}=40$).

As shown in Figure 9, with increases in node number, the packet delivery ratios of the four protocols also gradually increase and approach a stable value. When the network is relatively dense (n≥100), the packet delivery ratios of the four protocols above show little difference, owing to the low probability of the routing void. However, the FLGR protocol performs better in the sparse network (n≤60). This improvement is due to more void nodes occurring in the sparse network, and the FLGR protocol can thus effectively discard the void node through the routing void avoidance scheme. Although the routing void problem can be solved in the other three protocols, the transmission delay vastly increases because of the location server algorithm. As a result, the packet loss ratios of the GPSR, GPSR-AD, and LPESG protocols are higher than that of the FLGR protocol.

**Figure 10:**
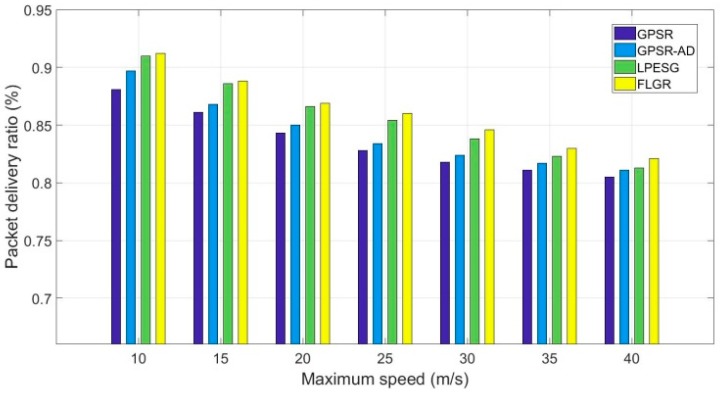
Impact of maximum speed on packet delivery ratio with n=60.

Figure 10 shows the impact of maximum speed on the packet delivery ratio in a sparse network (n=60).

As shown in Figure 10, the packet delivery ratios of the four protocols decrease with the increase in maximum speed. In addition, the FLGR and LPESG protocols perform better than the GPSR and GPSR-AD protocols in a slowly changing network ($v_{\max}\leq 30$). The packet delivery ratio of the FLGR protocol is higher than that of the others in a quickly changing network ($v_{\max}\geq 40$). This phenomenon occurs due to the following reasons: (1) Instability of the link increases with the increase in maximum speed, resulting in a decline of the packet delivery ratio. (2) In a slowly changing network ($v_{\max}\leq 30$), the routing void problem can be avoided by discarding the void node in the FLGR protocol, and it can be solved by adjusting the transmission power in the LPESG protocol. For the GPSR and GPSR-AD protocols, the peripheral forwarding mode is adopted when meeting the routing void problem, which can bring about the network planarization problem. (3) In a quickly changing network ($v_{\max}\geq 40$), the location information of destination node should be frequently obtained in the GPSR, GPSR-AD, and LPESG protocols, which effectively increases the transmission delay.

#### 4.2.2. Routing Overhead Ratio

The network routing overhead ratio refers to the ratio of routing packets to the total packets in the simulation process, and a lower routing overhead ratio indicates that the routing algorithm converges faster. In this paper, the routing overhead includes the cost of obtaining the location of the destination node.

**Figure 11:**
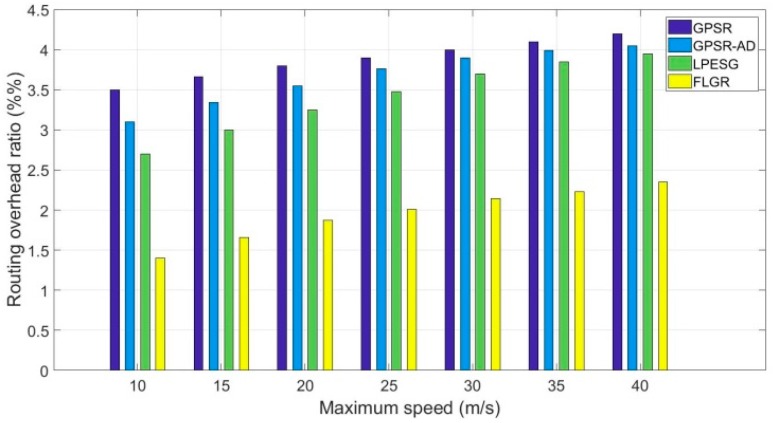
Impact of maximum speed on routing overhead ratio with n=140.

Figure 11 shows the impact of maximum speed on the routing overhead ratio in a dense network (n=140).

As shown in Figure 11, the routing overhead of the GPSR, GPSR-AD, LPESG, and FLGR protocols increases gradually with the increase in maximum speed. Compared with the GPSR, GPSR-AD, and LPESG protocols, the routing overhead of the FLGR protocol reduces significantly. This reduction occurs because in the former three protocols, the location information of the destination node should be obtained through the GLS algorithm, increasing the routing overhead. For the FLGR protocol, although the number of the routing hop is larger than that of the GPSR protocol (as shown in Figure 8), the GLS algorithm is not needed. Hence, the routing overhead decreases considerably, compared with the other protocols.

**Figure 12:**
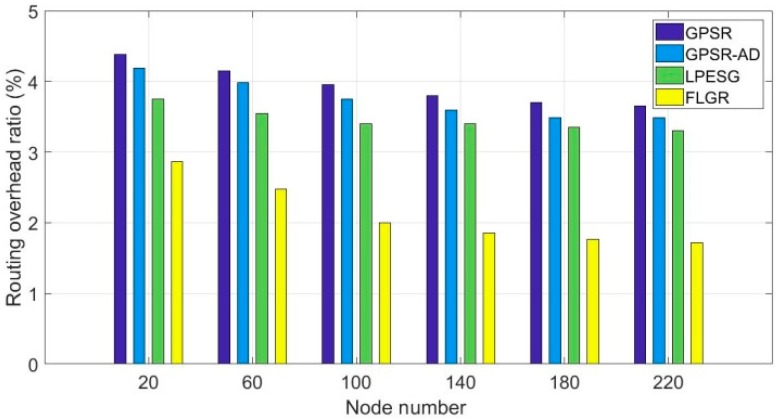
Impact of node number on routing overhead ratio with $v_{\max}=20$.

Figure 12 shows the impact of node number on the routing overhead ratio in a slowly changing network ($v_{\max}=20$).

As shown in Figure 12, the routing overhead ratios of the four protocols decrease with the increases in node number. Compared with GPSR, GPSR-AD, and LPESG protocols, the routing overhead ratio of the FLGR protocol is significantly reduced. Although the packet delivery ratios of the LPESG and FLGR protocols are almost the same in a slowly changing network (as shown in Figure 10), the LPESG needs additional routing overhead through GLS algorithm, as is the case with the GPSR and GPSR-AD protocols.

### 4.3. Complexity Analysis

Judging from the perspective of time complexity, according to the routing process of the FLGR protocol, all the fuzzy sets of the parameters should be traversed once. Assuming there are u CNs of one EN, and the number of node parameters participating in decision making is m, the time complexity of the comprehensive evaluation fuzzy set obtained is o(mu). The time complexity of the defuzzification is o(u). The time complexity of the ranking is o(ulog(u)). Therefore, the total time complexity is o(mu).

## 5. Conclusions

The geographic routing protocol can establish routing quickly and reliably based on the location information of neighbor nodes and destination nodes. However, additional routing overhead is incurred, because the location information of destination nodes needs to be obtained in real-time through the location service algorithm. In addition, the existing geographic routing protocols also have the problem of routing void and inaccurate positioning.

To solve all the above problems, based on the fuzzy logic system, the FLGR protocol has been put forward without the real-time location information of the destination node. Aiming for fewer hops and a higher packet delivery rate, the selection criteria and parameters for assessment have also been proposed.

In addition, the routing void problem was too considered in the FLGR protocol. The routing void avoidance scheme was proposed to reduce the rate of routing failure by adjusting the CNR and discarding the void node. The forwarding node can be selected through the fuzzification, fuzzy reasoning, and defuzzification of node parameters.

Finally, the feasibility of the FLGR forwarding mode was verified and the performance of FLGR protocol analyzed via simulation. Simulation results show that the proposed FLGR forwarding mode is able to effectively avoid the routing void problem. Compared with existing protocols, the FLGR protocol has lower routing overhead and a higher packet delivery rate in a sparse network.

## Figures and Tables

**Table 1:** Detailed list of acronyms and notations.

Acronyms and Notations	Meanings
EN	Election node
CNR	Candidate node region
CN	Candidate node
$n_{i}$	Node i
$l_{i}$	Location information of $n_{i}$
N	Number of nodes in network
S	Area of network region
$v_{\max}$	Maximum speed of node
R	Transmission radius of node
$t_{ij}$	Time interval from the previous update time of $n_{j}$ to the current moment according to the local network location table of $n_{i}$
$r_{ij}$	Fuzzy location region radius of destination node $n_{j}$ according to $t_{ij}$
$\theta_{ij}^{aero}$	Angle of the fuzzy location region according to $r_{ij}$
$D_{ij}$	Distance between $n_{i}$ and $n_{j}$
$\rho_{kj}$	Density of nodes in the CNR of CN $n_{k}$ (destination node $n_{j}$)
$\rho_{global}$	Density of nodes in network
$\eta_{kj}$	Relative density between $\rho_{kj}$ and $\rho_{global}$
$\Omega_{kj}$	Distribution degree of nodes in the CNR of CN $n_{k}$ (destination node $n_{j}$)
$L^{opti\_kj}$	Optimal locations of nodes in the CNR of CN $n_{k}$ (destination node $n_{j}$)
μ(x)	Gaussian subordinating degree function
E	Comprehensive assessment of CN

**Table 2:** Format of Hello message.

Parameters	Values
Num_seq	2 Bytes
Hello message ID	2 Bytes
Node location	16 Bytes
Neighbor nodes IDs	2 Bytes * num_neighbor
Neighbor nodes locations	16 Bytes * num_neighbor

**Table 3:** Format of data message.

Parameters	Values
Num_seq	2 Bytes
Data message ID	2 Bytes
Destination node ID	2 Bytes
Destination node location	16 Bytes
Update time of destination node	2 Bytes
Node IDs_pre	2 Bytes * Num_pre
Nodes locations_pre	16 Bytes * Num_pre
Data	1280 Bytes

**Table 4:** Local network location table.

Parameters	Values
Node ID	2 Bytes
Node location	16 Bytes
Update time	2 Bytes

**Table 5:** Part of fuzzy reasoning rule base.

ID	IF			THEN
	D	η	Ω	*η*
1	near	low	bad	very low
2	middle	middle	middle	middle
3	far	high	excellent	very high
…	…	…	…	…

**Table 6:** Defuzzification table of parameter E.

Fuzzy Sets	Values
very low	0.03133
low	0.25
medium	0.5
high	0.75
very high	0.9687

**Table 7:** Network parameters in simulation.

Parameters	Values
Network simulation	10 min.
Network node number	20, 60, 100, 140, 180, 220
Application	CBR for UDP
Maximum speed	10, 15, 20, 25, 30, 35, 40 m/s
Packet size	1280 bytes
Propagation delay	1 μs
Channel bit rate	1 Mbps
Frequency of Hello message	1 Hz, 1.5 Hz, 2 Hz, 2.5 Hz, 3 Hz, 3.5 Hz and 4 Hz
Maximum Num_pre	10

## Appendix A

**Table A1:** Fuzzy reasoning rule base.

ID	IF			THEN
	D	*η*	Ω	E
1	near	low	bad	very low
2	near	low	middle	very low
3	near	low	excellent	low
4	near	middle	bad	very low
5	near	middle	middle	low
6	near	middle	excellent	middle
7	near	high	bad	low
8	near	high	middle	middle
9	near	high	excellent	high
10	middle	low	bad	very low
11	middle	low	middle	low
12	middle	low	excellent	middle
13	middle	middle	bad	low
14	middle	middle	middle	middle
15	middle	middle	excellent	high
16	middle	high	bad	middle
17	middle	high	middle	high
18	middle	high	excellent	very high
19	far	low	bad	low
20	far	low	middle	middle
21	far	low	excellent	high
22	far	middle	bad	middle
23	far	middle	middle	high
24	far	middle	excellent	veryhigh
25	far	high	bad	high
26	far	high	middle	very high
27	far	high	excellent	very high

## References

[1] Akyildiz I.F., Su W., Sankarasubramaniam Y., Cayirci E. (2002). Wireless sensor networks: A survey. Comput. Netw..

[2] Rawat P., Singh K.D., Chaouchi H. (2014). Wireless sensor networks: A survey on recent developments and potential synergies. J. Supercomput..

[3] Bhatti G. (2018). Machine Learning Based Localization in Large-Scale Wireless Sensor Networks. Sensors.

[4] Wang K., Chen P., Teunissen P. (2018). Fast Phase-Only Positioning with Triple-Frequency GPS. Sensors.

[5] Li J., Jannotti J., De Couto D.S.J. A scalable location service for geographic ad hoc routing. Proceedings of the 6th Annual International Conference on Mobile Computing and Networking.

[6] Luo K., Wang J.X., Zhao X.N. (2008). Survey on geographic routing in wireless sensor networks. Comput. Sci..

[7] Karp B., Kung H.T. GPSR: Greedy perimeter stateless routing for wireless networks. Proceedings of the 6th Annual International Conference on Mobile Computing and Networking.

[8] Zadeh L.A. (1996). Fuzzy Sets, Fuzzy Logic, and Fuzzy Systems.

[9] Pedrycz W., Gomide F. (2007). Fuzzy Systems Engineering: Toward Human-Centric Computing.

[10] Li D.Q., Liu H.Y., Cao Q.G., Wang H.C. (2009). New routing algorithm based on geographical location: GPSR-AD. J. Comput. Appl..

[11] Guan W., Ren Q.H., Zhang H.Y., Zheng B. (2011). Improvement of greedy forwarding schemes based on fuzzy logic control. Appl. Res. Comput..

[12] Park J., Kim Y.N., Byun J.Y. A forwarder selection method for greedy mode operation of a geographic routing protocol in a WSN. Proceedings of the 2013 Fifth International Conference on Ubiquitous and Future Networks (ICUFN).

[13] Yu H., Ahn S. A geographic routing scheme with dead-end avoidance for large-scale MANETs. Proceedings of the 2015 International Conference on Information and Communication Technology Convergence (ICTC).

[14] Sa Y., Li N., Zhang L.L., Zhu L.C., Zhang Z.W. (2015). Routing algorithm for Ad Hoc networks based on location prediction. J. Chin. Comput. Syst..

[15] Sa Y., Zhu Z.Q., Zhu L.C., Zhang Z.W. (2015). High performance routing algorithm based on geographic location prediction. J. Northeast. Univ. (Nat. Sci.).

[16] Wang Z.C., Hou H.H., Lian R. (2018). Geographic routing algorithm based on location prediction in WSN. Comput. Sci..

[17] Lin J.L., Kuo G.S. A novel location-fault-tolerant geographic routing scheme for wireless ad hoc networks. Proceedings of the IEEE 63rd Vehicular Technology Conference, VTC 2006-Spring.

[18] Xiao H., Zhang H., Wang Z. An RSSI based DV-hop algorithm for wireless sensor networks. Proceedings of the 2017 IEEE Pacific Rim Conference on Communications, Computers and Signal Processing (PACRIM).

[19] Khan S., Ahmad M.I., Hussain F. (2018). Exponential utility function based criteria for network selection in heterogeneous wireless networks. Electron. Lett..

[20] He Y., He Z. (2016). Extensions of Atanassov’s Intuitionistic Fuzzy Interaction Bonferroni Means and Their Application to Multiple-Attribute Decision Making. IEEE Trans. Fuzzy Syst..

[21] Zhang X.G., Mahadevan S. (2017). A game theoretic approach to network reliability assessment. IEEE Trans. Reliab..

[22] Li G.C. (2000). Representation, ranking, and distance of fuzzy number with exponential membership function using graded mean integration method. Tamsui Oxf. J. Math. Sci..

